# Basal cell carcinoma overlying a dermatofibroma in an African American patient

**DOI:** 10.1016/j.jdcr.2024.06.009

**Published:** 2024-06-22

**Authors:** Simone Botts, Constance Ediale, Valerie D. Callender

**Affiliations:** a Georgetown University School of Medicine, Washington, District of Columbia; b Department of Dermatology, Howard University College of Medicine, Washington, District of Columbia; c Callender Dermatology and Cosmetic Center, Glenn Dale, Maryland

**Keywords:** basal cell carcinoma, basal cell carcinoma over dermatofibroma, basal cell hyperplasia, dermatofibroma, neoplastic dermatofibroma, skin of color

## Introduction

Dermatofibromas (DFs) are common subcutaneous, firm nodules that often are asymptomatic and frequently present on the lower extremities; however, they can occur on any part of the body.^1^ Although DFs are generally benign, there have been very few reported cases of concurrent malignancy, specifically basal cell carcinomas (BCCs) arising within DFs.^2^, ^3^, ^4^, ^5^, ^6^, ^7^ This phenomenon is rare; however, changes in the epidermis overlying DFs are observed in nearly 80% of cases, spanning from basic hyperplasia to the proliferation of basaloid cells, which can appear clinically and morphologically similar to BCC.^3^ To our knowledge, there are no reports of these occurrences in skin of color. Here, we present a case of a BCC overlying a DF in an African American (AA) patient.

BCC is the most common form of skin cancer worldwide with the highest predominance in White, but is particularly rare in AA and darker skin types. The incidence of BCC in AA is ∼2% of all cancers.^8^ BCC is the second most common skin cancer in AA after squamous cell carcinoma.^8^ In skin of color, BCC generally presents as pigmented lesions in >50% of cases and predominantly presents on the head and neck (90% of cases).^8^ This can be challenging to identify due to deviation from typical features, and has contributed to later diagnoses and higher morbidity/mortality rates.^8^ The rarity of skin cancer diagnoses in AA is in part due to the overall low incidence of skin cancer in darker skin from some of melanin’s protective effect from UV rays, but also attributed to the current disparities in access to health care and gaps in understanding and diagnosing of skin cancer in darker skin.^8^ Therefore, this case highlights an important finding in skin of color.

## Case report

**Fig 1:**
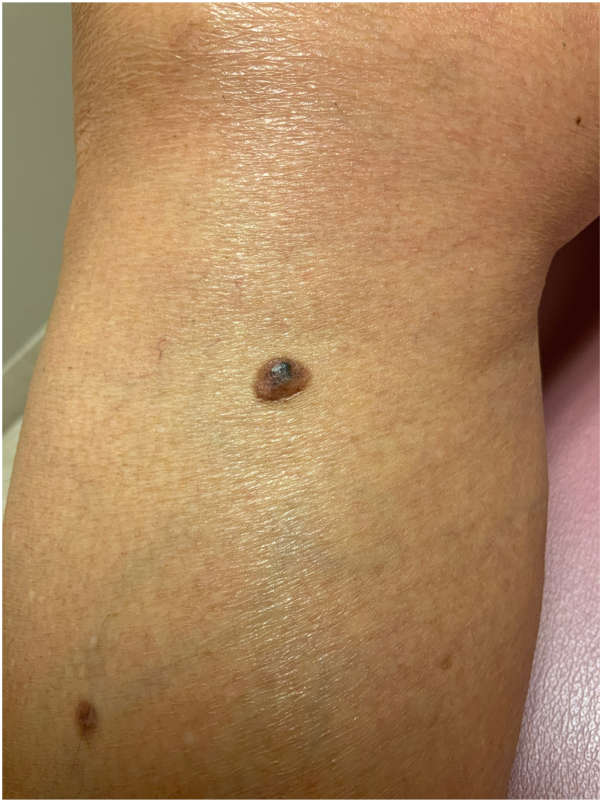
Dermatofibroma on patient's leg. Pigmented papule on patient’s leg with a dark central area consistent with a dermatofibroma. The lesion has typical characteristics of dermatofibroma, including central hyperpigmentation surrounded by slightly elevated, firm, and well-defined borders.

**Fig 2:**
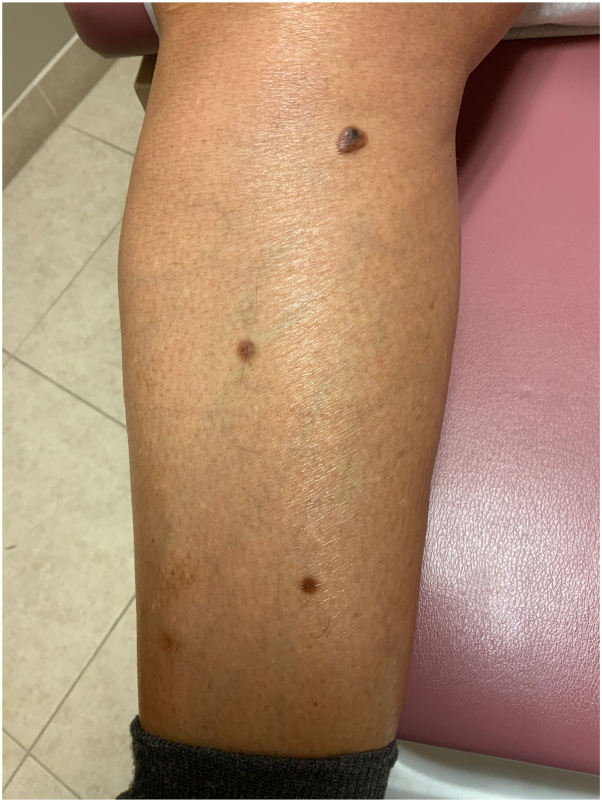
Dermatofibroma on patient's leg. Pigmented papule on patient’s leg with a dark central area consistent with a dermatofibroma. Also visible are other pigmented macules compared to the lesion of concern. The lesion has typical characteristics of dermatofibroma, including central hyperpigmentation surrounded by slightly elevated, firm, and well-defined borders.

**Fig 3:**
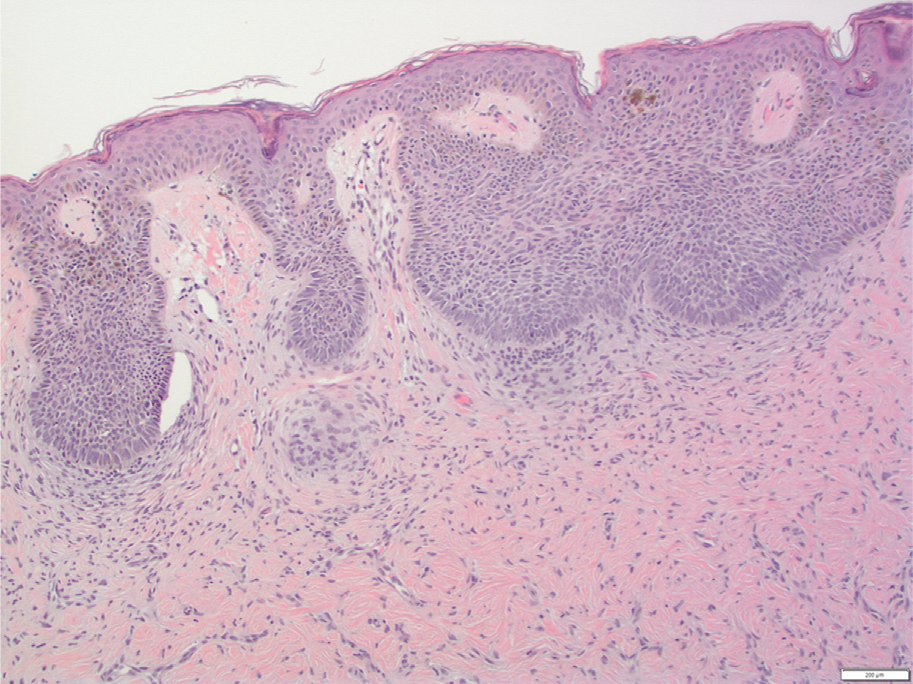
H&E-stained section of lesion. High power view of histopathological image showing basal cell proliferation consistent with basal cell carcinoma, underlying dermis with spindle cell proliferation and collagen trapping, and retraction artifacts.

**Fig 4:**
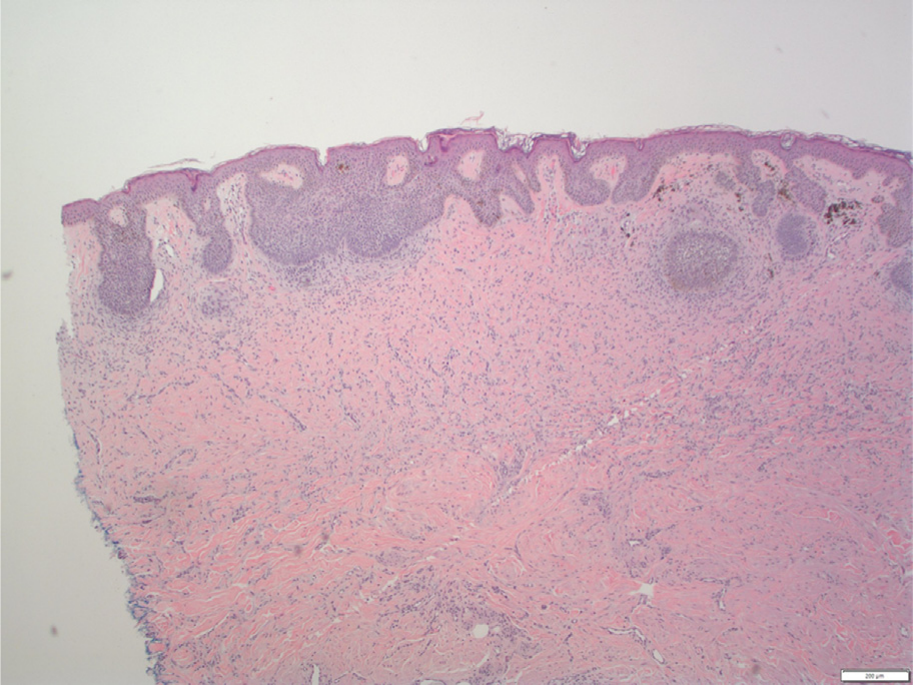
H&E-stained section of lesion. Lower power view of histopathological image showing overall lesion architecture with dermatofibroma in upper layers and basal cell proliferation consistent with basal cell carcinoma beneath it, with retrction artifact.

**Fig 5:**
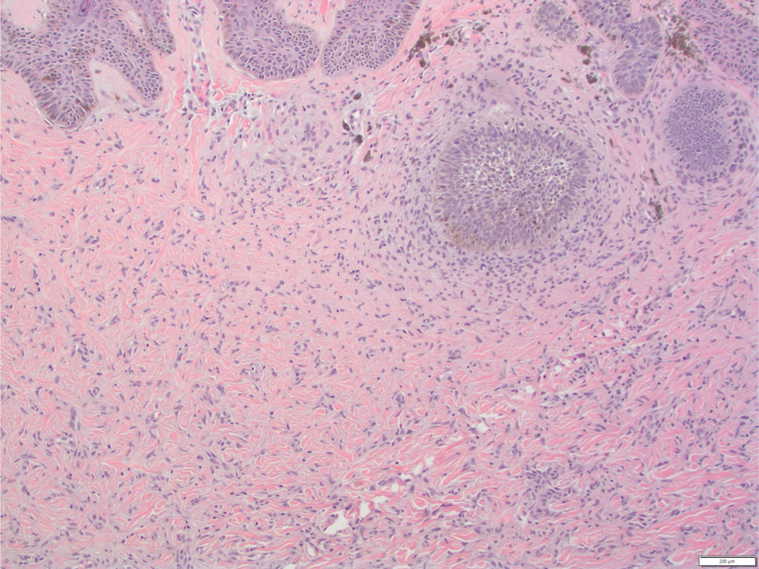
H&E stained-section of lesion. High power view of histopathological image showing basal cell proliferation consistent with basal cell carcinoma, underlying dermis with spindle cell proliferation, collagen trapping, and retraction artifacts.

A 71-year-old AA woman with no significant medical history presented with a red to dark brown papule on her lower portion of the right leg (Fig 1). On examination, there were multiple DFs on the lower extremities; however, the lesion of concern appeared to be irregularly pigmented (Fig 2). She reported that the lesion appeared when she was in her 20s and denied preceding trauma to the area. She denied any associated symptoms. A shave biopsy was performed. The microscopic examination results showed downward bud-like aggregates of polygonal basaloid cells attached to the epidermal undersurface, as well as peripheral palisading and cleft artifacts (Fig 3, Fig 4, Fig 5). Interlacing bundles of histiocytes and spindle cells were within the collagenous stroma of the dermis. Immunohistochemical stains were positive for p40 and Ber-Ep4 in the superficial basaloid cells and Factor XIIIa in the dermal spindle cells. These results were consistent with the diagnosis of a DF with overlying BCC given the presence of isolated basaloid lobules projecting from the lower margin of the epidermis and large basaloid lobules with peripheral nuclear palisade and retraction artifacts (Fig 3, Fig 4, Fig 5). All features that support a true BCC. Benign basaloid proliferation usually has minimal to no retraction artifacts.

Subsequently a month later, the patient underwent wide local excision for treatment. The patient tolerated this procedure well and returned for follow-up 6 months later. The excision healed well, and the patient reported no concerns. The patient was advised to follow up every 6 to 9 months.

## Discussion

In the current literature, only a few cases have reported true BCCs overlying DFs. This rarity is likely attributed to BCC-like changes in DFs, which typically lack malignant potential and more commonly represent benign induction occurrences, such as basaloid cell hyperplasia.^9^^,^^10^ Additionally, there are numerous histologic mimickers of BCCs with overlapping histologic features that pose challenges in differentiation. These include follicular induction (FI) over DF, benign hair-germ tumors (such as trichoepithelioma and trichoblastoma), tumor of the follicular infundibulum, squamous cell carcinoma, basaloid follicular hamartoma, microcystic adnexal carcinoma, sebaceous carcinoma, and Merkel cell carcinoma.^10^ Certain characteristics can differentiate BCC from mimickers. For example, FI may demonstrate follicular differentiation such as germinative buds and papillary mesenchymal bodies, and lacks enlarged crowded atypical nuclei, mitotic figures, or apoptotic bodies often seen in BCC.^10^ Although BCC lacks epidermal hyperplasia between nests. Also, Ber-Ep4 is an antibody often used to confirm BCC, whereas CK20+ in Merkel cells can confirm FI.^10^ Therefore, identifying key histologic features, and often immunochemistry, are crucial to differentiate and accurately diagnose true BCC, along with a comprehensive assessment of clinical presentation and morphology.

Previous case reports in the literature have predominantly detailed occurrences in lighter skin types, indicating that this phenomenon is exceedingly rare, particularly in skin of color.^2^, ^3^, ^4^, ^5^, ^6^, ^7^ The cases in the literature used histopathologic features to confirm the diagnosis of BCC over DF, noting FI and epidermal proliferation of basaloid cells, plus subtle features that confirmed overlying BCC such as nests, palisading cells, and retraction artifacts from the adjacent dermis. These specific histopathologic features distinguish a true BCC from BCC-like hyperplasia.^2^, ^3^, ^4^, ^5^, ^6^, ^7^ It is hypothesized that the inductive effect of DF and fibrohistiocytic proliferation may contribute to the development of BCC; however, further research is needed.^4^

Both BCCs and DFs can present as raised, firm nodules, with textures and coloration that may overlap, ranging from pink to brown. Although BCCs typically exhibit rolled or raised borders, both lesions can display concerning irregularities. Therefore, due to the numerous overlapping clinical features that can render DFs and BCC clinically indistinguishable, histopathologic examination is imperative for definitive diagnosis.^9^^,^^10^ Although rare, this case underscores the significance of utilizing skin biopsy to distinguish between benign DFs and those harboring malignant potential. Clinical indicators such as changes in size, irregularities in shape or color, or symptomatic lesions should prompt histopathologic investigation. Lesions of confirmed malignant nature should be excised by wide local excision, and benign-appearing lesions should be monitored for changes. Moreover, this case highlights the importance of recognizing diverse presentations across various skin tones and considering cutaneous malignancy despite its lower incidence in darker skin types. Continued education should be provided to allow for proper diagnosis in skin of color, as early diagnosis and prevention can greatly improve outcomes and patient care.

## Conflicts of interest

None disclosed.

## References

[1] Myers D.J., Fillman E.P. (2023). https://www.ncbi.nlm.nih.gov/books/NBK470538/.

[2] Goette D.K., Helwig E.B. (1975). Basal cell carcinomas and basal cell carcinoma-like changes overlying dermatofibromas. Arch Dermatol.

[3] Gulin S.J., Loncaric D., Rados J. (2020). Basal cell carcinoma overlying a dermatofibroma: a rare collision tumor. Dermatol Pract Concept.

[4] Córdoba S., Hernández A., Romero A. (2005). Carcinoma basocelular sobre dermatofibroma [Basal cell carcinoma overlying a dermatofibroma]. Article in Spanish. Actas Dermosifiliogr.

[5] Bryant J. (1977). Basal cell carcinoma overlying long-standing dermatofibromas. Arch Dermatol.

[6] Goette D.K. (1976). Basal cell carcinoma overlying dermatofibroma. Arch Dermatol.

[7] Mortimore A., Muir J. (2020). Basal cell carcinoma in dermatofibroma: dual diagnosis. Aust J Gen Pract.

[8] Davis D.S., Robinson C., Callender V.D. (2021). Skin cancer in women of color: epidemiology, pathogenesis and clinical manifestations. Int J Womens Dermatol.

[9] Lindboe C.F., Løvdal L. (2011). Epidermal basaloid cell hyperplasia overlying dermatofibromas. Am J Dermatopathol.

[10] Stanoszek L.M., Wang G.Y., Harms P.W. (2017). Histologic mimics of basal cell carcinoma. Arch Pathol Lab Med.

